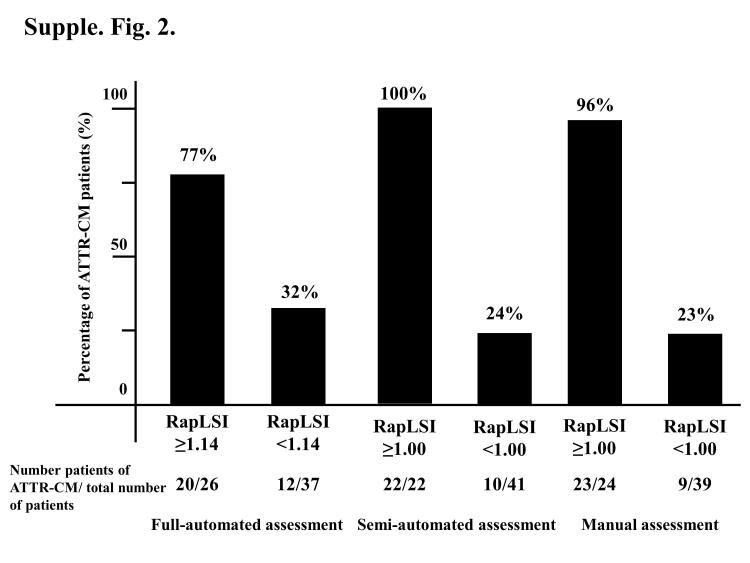# Usefulness of automatic assessment for longitudinal strain to diagnose wild-type transthyretin amyloid cardiomyopathy

**DOI:** 10.1016/j.ijcha.2023.101227

**Published:** 2023-06-22

**Authors:** Hiroki Usuku, Eiichiro Yamamoto, Daisuke Sueta, Kanako Imamura, Fumi Oike, Kyohei Marume, Masanobu Ishii, Shinsuke Hanatani, Yuichiro Arima, Seiji Takashio, Seitaro Oda, Hiroaki Kawano, Mitsuharu Ueda, Hirotaka Matsui, Kenichi Tsujita

**Affiliations:** a Department of Laboratory Medicine, Kumamoto University Hospital, Kumamoto, Japan; b Department of Cardiovascular Medicine, Graduate School of Medical Sciences, Kumamoto University, Kumamoto, Japan; c Center of Metabolic Regulation of Healthy Aging, Kumamoto University, Faculty of Life Sciences, Kumamoto, Japan; d Department of Diagnostic Radiology, Faculty of Life Sciences, Kumamoto University, Kumamoto, Japan; e Department of Neurology, Graduate School of Medical Sciences, Kumamoto University, Kumamoto; f Department of Molecular Laboratory Medicine, Faculty of Life Sciences, Kumamoto University, Kumamoto, Japan

**Keywords:** Transthyretin amyloid cardiomyopathy, Two-dimensional speckle tracking echocardiography, Relative apical longitudinal strain index, Automatic assessment

## Abstract

**Background:**

Left ventricular (LV) apical sparing by transthoracic echocardiography (TTE) has not been widely accepted to diagnose transthyretin amyloid cardiomyopathy (ATTR-CM), because it is time consuming and requires a level of expertise. We hypothesized that automatic assessment may be the solution for these problems.

**Methods-and-Results:**

We enrolled 63 patients aged ≥70 years who underwent ^99m^Tc-labeled pyrophosphate (^99m^Tc-PYP) scintigraphy on suspicion of ATTR-CM and performed TTE by EPIQ7G, and had enough information for two-dimensional speckle tracking echocardiography at Kumamoto University Hospital from January 2016 to December 2019. LV apical sparing was described as a high relative apical longitudinal strain (LS) index (RapLSI). Measurement of LS was repeated using the same apical images with three different measurement packages as follows: (1) full-automatic assessment, (2) semi-automatic assessment, and (3) manual assessment. The calculation time for full-automatic assessment (14.7 ± 1.4 sec/patient) and semi-automatic assessment (66.7 ± 14.4 sec/patient) were significantly shorter than that for manual assessment (171.2 ± 59.7 sec/patient) (p < 0.01 for both). Receiver operating characteristic curve analysis showed that the area under curve of the RapLSI evaluated by full-automatic assessment for predicting ATTR-CM was 0.70 (best cut-off point; 1.14 [sensitivity 63%, specificity 81%]), by semi-automatic assessment was 0.85 (best cut-off point; 1.00 [sensitivity, 66%; specificity, 100%]) and by manual assessment was 0.83 (best cut-off point; 0.97 [sensitivity, 72%; specificity, 97%]).

**Conclusion:**

There was no significant difference between the diagnostic accuracy of RapLSI estimated by semi-automatic assessment and that estimated by manual assessment. Semi-automatically assessed RapLSI is useful to diagnose ATTR-CM in terms of rapidity and diagnostic accuracy.

## 1 Introduction

Amyloid cardiomyopathy is a progressive infiltrative cardiomyopathy characterized by restrictive cardiomyopathy and various arrhythmias secondary to infiltration of the conduction system [1]. The two main types of amyloid cardiomyopathy are amyloid light-chain amyloidosis and transthyretin (TTR) amyloidosis (ATTR). ATTR is further classified into two subtypes based on the presence or absence of a genetic mutation: variant ATTR and wild-type ATTR (ATTRwt). Transthyretin amyloid cardiomyopathy (ATTR-CM) is becoming increasingly recognized because of population aging, advancements in the understanding of the disease pathobiology, and the potential benefits of emerging therapies [2].

The hallmarks of amyloid cardiomyopathy on transthoracic echocardiography (TTE) are increased left ventricle (LV) thickness, left atrial (LA) enlargement, and reduced systolic and diastolic LV function [3], [4]. However, these findings can also be present in other heart diseases with increased cardiac afterload. LV apical sparing, which is described as a high relative apical longitudinal strain (LS) index (RapLSI), is a pattern of regional differences in deformation in which the LS in the basal and middle segments of the LV is more severely impaired than that in the apical segments [5]. LV apical sparing is highly specific for amyloid cardiomyopathy and has incremental diagnostic value over other echocardiographic parameters traditionally used for this purpose [6]. Despite this evidence, LV apical sparing is not yet a part of the clinical routine, because measurement of LS is time consuming and requires a level of expertise [7], [8]. Thus, we hypothesized that the reliable automation of LS measurement could solve both of these problems, and the aims of the present study were to clarify the feasibility, reproducibility and diagnostic accuracy of RapLSI calculated by automatic assessment compared with that calculated by standard manual assessment.

## 2 Methods

### 2.1 Study population

149 consecutive patients aged ≥ 70 years were referred to Kumamoto University Hospital on suspicion of ATTR-CM, and underwent ^99m^Tc-labeled pyrophosphate (^99m^Tc-PYP) scintigraphy from January 2016 to December 2019. Of these, we selected 78 patients whom TTE was performed by EPIQ7G. Next, we excluded 15 patients because 8 patients had insufficient information for evaluation by two-dimensional speckle tracking echocardiography,1 patient who was diagnosed as AL amyloidosis, and 6 patients who could not be diagnosed as ATTR-CM in ^99m^Tc-PYP scintigraphy positive group. Therefore, 63 patients were enrolled in our present study (Supple. Fig. 1). Baseline clinical characteristics, laboratory findings and TTE data at diagnostic period were obtained while the patients were in a clinically stable condition.

This study conformed to the principles outlined in the Declaration of Helsinki. It was approved by the institutional review board and ethics committees of Kumamoto University (No. 1588). The requirement for informed consent was waived because of the low-risk nature of this retrospective study and the inability to obtain consent directly from all patients. Instead, we extensively announced this study protocol at Kumamoto University Hospital and on our website (https://www2.kuh.kumamoto-u.ac.jp/tyuokensabu/index.html) and gave patients the opportunity to withdraw from the study.

### 2.2 Conventional echocardiographic measurements

Conventional echocardiographic parameters by TTE were examined in enrolled patients in stable condition using the same equipment (EPIQ 7G; Philips, Bothell, WA, USA), which were equipped with a 2.5-MHz phased-array transducer. In patients with atrial fibrillation, we used averaged beats in three to five consecutive cardiac cycles as representative beats to evaluate echocardiographic analysis. The chamber size, LA volume index (LAVI), LV wall thickness, LV ejection fraction, and rate between peak early diastolic velocity of LV inflow (E velocity) and peak early diastolic velocity on the septal corner of the mitral annulus (e’) (E/e’ ratio) were evaluated using standard procedures [9], [10]. The peak early velocity of LV inflow (E velocity) and the peak early velocity on the septal corner of the mitral annulus (e’) were measured in the apical four-chamber view. Moderate or severe valvular diseases according to American heart association or American society of echocardiography (ASE) guideline were defined as valvular diseases in this study [11], [12]. To minimize bias, the echocardiography reviewers were blinded to the patients’ clinical history and data.

### 2.3 Two-dimensional speckle tracking echocardiography

Two-dimensional speckle tracking echocardiography was performed by two operator who was blinded to the clinical data and different from the operator who performed the conventional echocardiography. The regional LS, calculated from the echocardiography images in the 4-, 3-, and 2-chamber apical views, was determined in 16 or 18 segments of the LV in accordance with the ASE guideline [9]. The RapLSI was estimated as the hallmark of cardiac amyloidosis on echocardiography [5]. This index was calculated as [average apical LS/ (average basal LS + average mid LS)]. Strain was described in absolute values. Measurement of LS was repeated using the same apical images on three different measurement packages as follows.

1. Full-automatic assessment: full-automatic assessed LS was measured using Auto-Strain (TomTec-Arena, TomTec Imaging Systems, Unterscheleis-sheim, Germany). After setting the three apical views on the workspace, the investigator just informed the package of the 2-, 3, and 4-chamber views required for analysis. The endocardial border was automatically tracked and LS computed without manual adjustment from investigator (Fig. 1a).

**Fig. 1:**
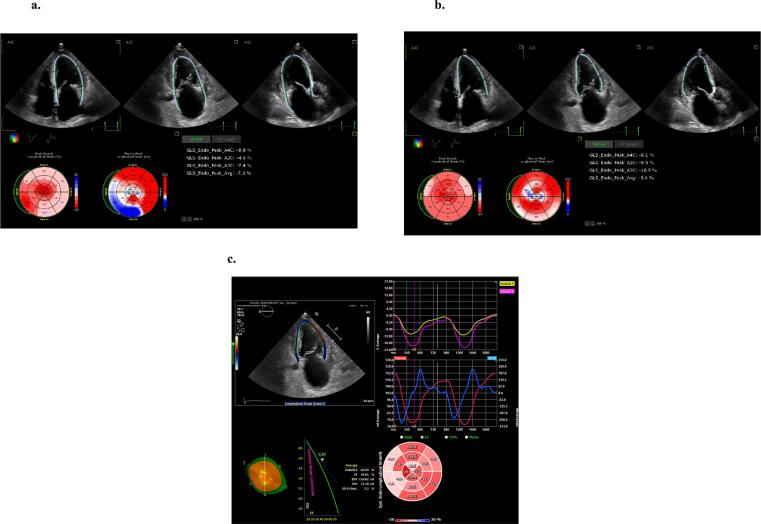
Representative case of full-automatic (a), semi-automatic (b) and manual (c) assessed LS. This was representative case of poor automatic assessment by LS. Automated trace lines were not drawn along the true endocardial border (a). Therefore, investigator manually corrected borders for semi-automatic assessment (b).
2. Semi-automatic assessment: after the automatic calculation of LS, the investigator manually corrected borders (Fig. 1-b).
3. Manual assessment: LS was assessed using two-dimensional cardiac performance analysis (2D-CPA): a manual measurement package (TomTec-Arena, TomTec Imaging Systems, Unterscheleis-sheim, Germany) using the defined apical 2-, 3-, and 4-chamber views for measurement. Three sampling points were manually identified at the septal and lateral mitral annulus and at the apical endocardium, and the endocardial borders were traced semi-automatically at the end of systole. After tracing the LV endocardial border, the dedicated software automatically tracked the myocardium throughout the cardiac cycle. All tracking was reviewed and manually corrected if needed (Fig. 1-c).

The investigator performing the manual assessed LS was blinded to the results of the full-automatic and semi-automatic assessed LS. The time required for each LS assessment method were measured for all enrolled patients. We defined LS calculation time as the time from the initial selection of three apical views to computation of LS of 16 or 18 segments with each assessment method.

### 2.4 Intra-observer and Inter-observer variability

Intra- and inter-observer variability of RapLSI for each LS assessment were evaluated in the same images in all enrolled patients. Intra-observer variability was calculated between the first and second measurements (after 30 days) by the same investigator, and the investigator was blinded to the previous measurement. Inter-observer variability was calculated between the first measurements of two independent investigators, and both investigators were blinded to each result. Both investigators had enough experience for manual assessed LS, but had little experience for full- or semi-automatic assessment of LS.

### 2.5 ^99m^Tc-PYP scintigraphy

^99m^Tc-PYP scintigraphy was performed using a dual-head single-photon emission computed tomography (SPECT) / CT (Symbia T16; Siemens Healthcare, Erlangen, Germany) with low-energy high-resolution collimators. All patients were scanned 3 h after an intravenous injection of 370–740 MBq of ^99m^Tc-PYP (Fujifilm RI Pharma, Tokyo, Japan). Anterior and lateral thoracic planar views were obtained over 3  min duration. The acquisition parameters used for planar imaging were 256 × 256 matrix with 1.23 zoom factor. With the same system, thoracic SPECT images were acquired for each patient immediately after the planar scan. Cardiac accumulation of ^99m^Tc-PYP in all patients was assessed by a board-certified nuclear medicine radiologist. On planar images, myocardial tracer uptake was evaluated with a visual scoring method (0 = no myocardial uptake; 1 = myocardial uptake less than rib uptake; 2 = myocardial uptake equal to rib uptake; and 3 = myocardial uptake greater than rib uptake). Myocardial uptake was also analyzed quantitatively based on the heart-to-contralateral ratio (H/CL ratio) of the total counts in a region of interest over the heart, which was divided by the background count of a copied and mirrored region of interest over the contralateral chest. ^99m^Tc-PYP positivity was based on a visual grade 2 or 3 uptake and/or the H/CL ratio of ≥ 1.3 [13], [14], [15], [16].

### 2.6 Diagnosis of ATTR-CM

According to Japanese guideline [17], ATTR-CM was defined as having a cardiac biopsy specimen showing TTR amyloid deposits; or the combined findings of ^99m^Tc-PYP scintigraphy-positive and the absence of a monoclonal protein in serum or urine.

### 2.7 Statistical analysis

Continuous variables are presented as mean ± standard deviation. Nonnormally distributed variables are presented as medians (IQR). Categorical values are presented as number (percentage). The clinical characteristics were compared between ATTR-CM group and non ATTR-CM group using student’s *t*-test or the chi-squared test. Nonnormally distributed variables between these groups were compared by Mann-Whitney *U* test. We used the one way repeated measured analysis of variance (ANOVA) for comparisons of time after image selection between each method. We used Bonferroni test to perform pairwise comparisons for time after image selection. The intra-observer and inter-observer variability for the RapLSI evaluated by each method were assessed using the intraclass correlation coefficient (ICC). Pearson correlation coefficient was performed to evaluate the correlation between RapLSI assessed by each method. The agreement between each assessment was assessed using Bland-Altman analysis to quantify a systemic difference between two different methods. Receiver operating characteristic (ROC) curve analysis was used to compare the ability of RapLSI calculated by each method to evaluate ^99m^Tc-PYP scintigraphy positivity. Decision curve analysis (DCA) was used to incorporate the clinical consequences of a decision into evaluations of diagnostic tests or prediction models [18]. Statistical analyses were conducted with SPSS for Windows software, version 24.0 (IBM Corp., Armonk, NY, USA) and R program version 4.0.5 (package “PredictABEL”, R Foundation, Vienna, Austria). Statistical significance was defined as p < 0.05.

## 3 Results

### 3.1 Study population

In enrolled 63 patients, 32 patients (51%) were divided into ATTR-CM group and 31 patients (49%) were divided into non ATTR-CM group.

In the ATTR-CM group, all 32 patients were positive for ^99m^Tc-PYP scintigraphy. Of these, 24 patients had TTR amyloid deposition in the heart and the other 8 patients were diagnosed as ATTR-CM on the basis of the combined findings of ^99m^Tc-PYP scintigraphy-positive and the absence of a monoclonal protein in serum or urine. DNA analysis was performed for 28 patients in the ATTR-CM group; 26 patients had no TTR mutation (wild-type [ATTRwt]), and 2 patients had a TTR mutation (Val30Met mutation). In non ATTR-CM group, all 31 patients were negative for ^99m^Tc-PYP scintigraphy. In this group, 5 patients underwent tissue biopsy and no patients had amyloid deposition. In the non ATTR-CM group, 14 patients (45%) had aortic stenosis, 6 patients (19%) had hypertrophic cardiomyopathy, 1 patient had cardiac sarcoidosis and 10 patients could not classify to specific cardiac diseases.

### 3.2 Comparison of clinical characteristics between ATTR-CM group and non ATTR-CM group

Supplementary Table 1 shows the baseline clinical characteristics, laboratory findings and TTE findings between ATTR-CM group and non ATTR-CM group. In the baseline clinical characteristics, ATTR-CM group were significantly younger (77.2 ± 4.9 vs. 81.4 ± 6.12 years, p < 0.05), had higher rate of male sex (88% vs. 65%), higher BMI (23.0 ± 2.7 vs. 21.4 ± 3.2 kg/m^2^, p < 0.01) than non ATTR-CM group. In the laboratory findings, hemoglobin level was significantly higher in the ATTR-CM group than those in the non ATTR-CM group (13.7 ± 1.9 vs. 11.6 ± 1.8 g/dl, p < 0.01). In the conventional TTE findings, interventricular septum thickness in diastole (IVSTd) and left ventricular posterior wall thickness in diastole (LVPWTd) were significantly higher, and LVEF was significantly lower in the ATTR-CM group than in the non ATTR-CM group (IVSTd, 15.1 ± 2.2 vs. 12.9 ± 2.7 mm, p < 0.01, LVPWTd, 15.3 ± 3.0 vs. 12.2 ± 1.7 mm, p < 0.01, LVEF, 51.5 ± 11.2 vs. 58.6 ± 8.3, p < 0.01).

### 3.3 Calculation time for LS

**Fig. 2:**
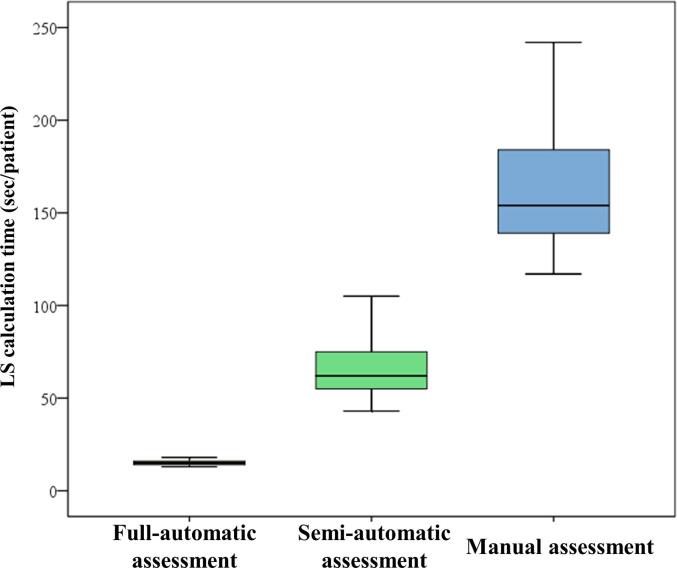
The average calculation time for each LS assessment. We defined LS calculation time as the time from the initial selection of three apical views to computation of LS of 16 or 18 segments with each assessment method. LS, longitudinal strain.

The average calculation time for each LS assessment is shown in Fig. 2. The calculation time for full-automatic assessment (14.7 ± 1.4 sec/patient) and that for semi-automatic assessment (66.7 ± 14.4 sec/patient) were both significantly shorter than that for manual assessment (171.2 ± 59.7 sec/patient) (p < 0.01 for both). In addition, the calculation time for full-automatic assessment was significantly shorter than that for semi-automatic assessment (p < 0.01).

### 3.4 Reproducibility of semi-automatic assessed RapLSI and manual assessed RapLSI

The intraclass correlations (ICC) for intra-observer reproducibility of semi-automatic assessed RapLSI and manual assessed RapLSI were 0.78 (95% CI 0.66–0.86) and 0.87 (95% CI 0.80–0.92), respectively. The ICC for inter-observer reproducibility of semi-automated assessed RapLSI and manual assessed RapLSI were 0.88 (95% CI 0.80–0.92) and 0.88 (95% CI 0.80–0.92).

### 3.5 Comparison between RapLSI assessment using different methods

The mean value of full-automatic assessed RapLSI was 1.18 ± 0.86, compared with 0.97 ± 0.44 for semi-automatic assessed RapLSI and 0.92 ± 0.46 for manual assessment. The spread of measurement of RapLSI estimated by semi-automatic assessment and manual assessment was similar and different from that estimated by full-automatic assessment.

**Fig. 3:**
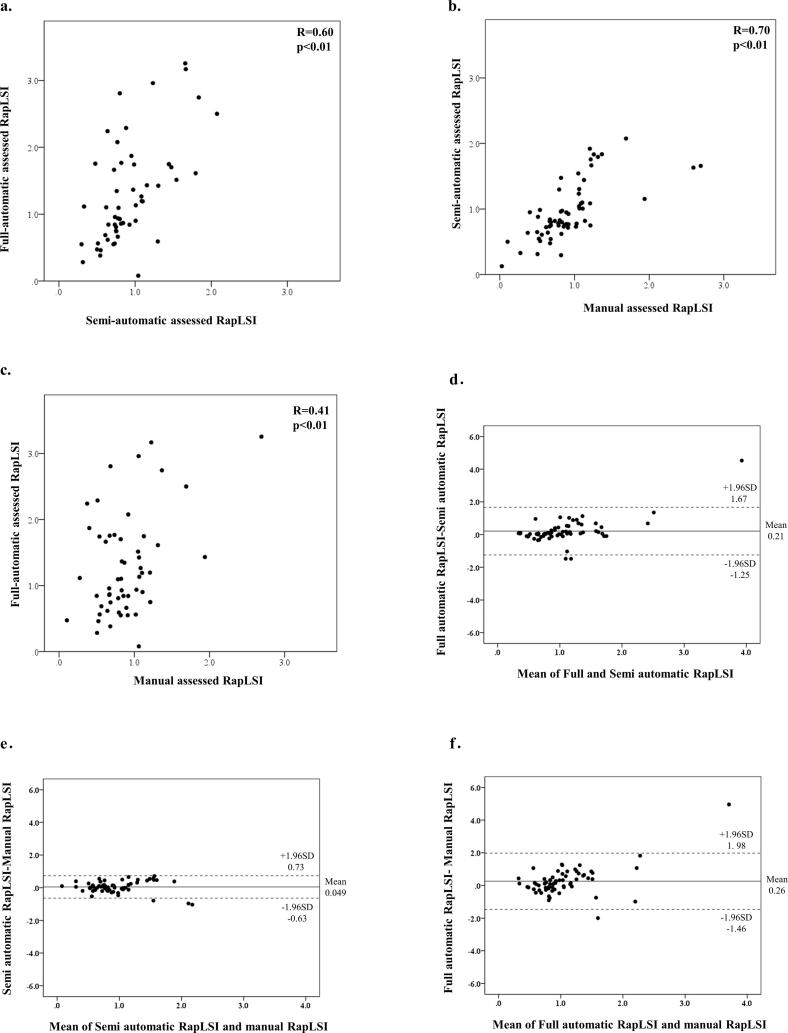
Comparisons between RapLSI assessments with scatterplot (a, b, c) and Bland-Altman plot (d, e, f). (a, d) semi-automatic vs. full-automatic assessed RapLSI, (b, e) manual vs. semi-automatic assessed RapLSI, (c, f) manual vs. full-automatic assessed RapLSI.

Pearson correlation coefficient and Bland-Altman analysis revealed that moderate correlation between semi-automatic assessed RapLSI and full-automatic assessed RapLSI (r = 0.60, p < 0.01, Fig. 3-a and 3-d), good correlation between semi-automatic assessed RapLSI and manual assessed RapLSI (r = 0.70, p < 0.01, Fig. 3-b and 3-e), and weak correlation between full-automatic assessed RapLSI and manual assessed RapLSI (r = 0.41, p < 0.01, Fig. 3-c and 3-f).

### 3.6 Receiver operating characteristic analysis and decision curve analysis

**Fig. 4:**
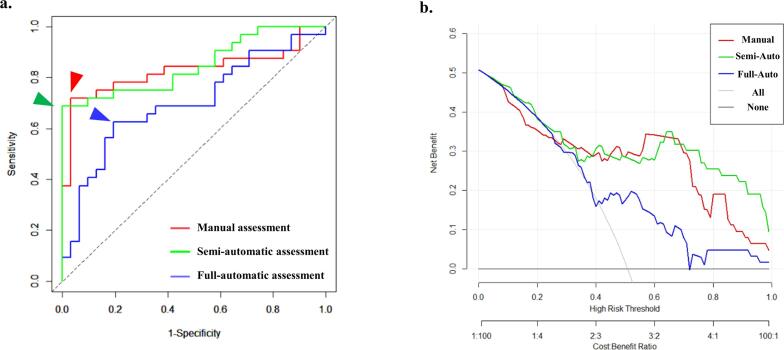
ROC analysis for the ability of full-automatic (blue line), semi-automatic (green line) and manual (red line) assessed RapLSI to predict ATTR-CM (a). AUC of RapLSI evaluated by full-automatic assessment was 0.70 and the best cut-off value of RapLSI was 1.14 (sensitivity, 63%; specificity, 81%) (blue arrow). The AUC of the RapLSI evaluated by semi-automatic assessment was 0.85 and the best cut-off value of RapLSI was 1.00 (sensitivity, 66%; specificity, 100%) (green arrow). The AUC of the RapLSI evaluated by manual assessment was 0.83 and the best cut-off value of RapLSI was 0.97 (sensitivity, 72%; Specificity, 97%) (red arrow). Decision curves for RapLSI evaluated by full-automatic assessment, semi-automatic assessment and manual assessment to predict ATTR-CM (b). The black line is the net benefit of treating no patients. The grey line is the net benefit of treating all patients. The blue line is the net benefit of treating patients according to RapLSI evaluated by full-automatic assessment. The green line is the net benefit of treating patients according to RapLSI evaluated by semi-automatic assessment. The red line is the net benefit of treating patients according to RapLSI evaluated by manual assessment. ROC, Receiver operating characteristic; RapLSI, relative apical longitudinal strain index; ATTR-CM, transthyretin amyloid cardiomyopathy; AUC, area under curve. (For interpretation of the references to colour in this figure legend, the reader is referred to the web version of this article.)

ROC analysis was performed to evaluate the difference of diagnostic utility to predict ATTR-CM between three assessment methods and to determine the cut-off value of RapLSI (Fig. 4-a). The area under curve (AUC) of the RapLSI evaluated by full-automatic assessment was 0.70 (blue line), and the best cut-off value of RapLSI was 1.14 (sensitivity, 63%; specificity, 81%, blue arrow). The AUC of the RapLSI evaluated by semi-automatic assessment was 0.85 (green line), and the best cut-off value of RapLSI was 1.00 (sensitivity, 66%; specificity, 100%, green arrow). The AUC of the RapLSI evaluated by manual assessment was 0.83 (red line), and the best cut-off value of RapLSI was 0.97 (sensitivity, 72%; Specificity, 97%, red arrow). The AUC of the RapLSI evaluated by full-automatic assessment was significantly lower than that evaluated by semi-automatic assessment (p = 0.02) and tended to be lower than that evaluated by manual assessment (p = 0.14). There was no significant difference between the AUC of the RapLSI evaluated by Semi-automatic assessment and that evaluated by manual assessment (p = 0.69).

Fig. 4-b demonstrated the decision curves for RapLSI evaluated by full-automatic assessment, semi-automatic assessment and manual assessment to predict ATTR-CM. Although RapLSI evaluated by full-automatic assessment was useful to predict ATTR-CM between threshold probabilities of 40–100%, RapLSI evaluated by semi-automatic assessment and by manual assessment were more useful than that evaluated by full-automatic assessment for most of the risk thresholds.

### 3.7 Difference of utility between full-automatic, semi-automatic and manual assessed RapLSI to diagnose ATTR-CM

Supplementary Fig. 2 showed the difference of predictive model for ATTR-CM between full-automatic, semi-automatic and manual assessment. When RapLSI was evaluated by full-automatic assessment, the rate of ATTR-CM in high RapLSI (≥1.14) group was 77% (20/26), and the rate of non ATTR-CM in low RapLSI (<1.14) group was 68% (25/37). When it was evaluated by semi-automatic assessment, the rate of ATTR-CM in high RapLSI (≥1.00) group was 100% (22/22), and the rate of non ATTR-CM in low RapLSI (<1.00) group was 76% (31/41). When it was evaluated by manual assessment, the rate of ATTR-CM in high RapLSI (≥0.97) group was 96% (23/24), and the rate of non ATTR-CM in low RapLSI (<0.97) group was 77% (30/39).

## 4 Discussion

In this study, we assessed the usefulness of automatic assessed RapLSI to predict ATTR-CM using novel automated software, and found the findings as follows: (1) there was no significant difference between the usefulness of RapLSI estimated by semi-automatic assessment and that estimated by manual assessment; (2) full-automatic and semi-automatic assessed LS can be performed in significantly shorter time than manual assessed LS; and (3) there were considerable discordances especially between full-automatic RapLSI and other measurement packages (semi-automatic and manual assessed RapLSI).

In these days, ATTR-CM is becoming increasingly recognized as common diseases especially in elderly patients, and approximately 13% of advanced-age patients who have heart failure with a preserved ejection fraction are reportedly diagnosed with ATTR-CM [19]. Several postmortem studies have revealed cardiac amyloid deposition in up to 25% of individuals over 80 years of age [20]. Although the number of newly diagnosed ATTR-CM dramatically increased [21], many ATTR-CM patients were thought to be still undiagnosed. Although a recent study showed that the transthyretin stabilizer tafamidis is associated with reductions in all-cause death cardiovascular related hospitalizations, and functional capacity and quality of life declines [22], the usefulness of tafamidis in advanced cases has not been clarified. To diagnose ATTR-CM in the early stage, LV apical sparing, which is described as a high RapLSI in the present study, is highly specific for amyloid cardiomyopathy and has incremental diagnostic value over other echocardiographic parameters traditionally used [5]. Because we previously reported that LV apical sparing was useful to diagnose ATTR-CM even in patients without typical findings of cardiac amyloidosis [23], aggressive usage of LV apical sparing was thought to be useful to prevent diagnostic delay. However, LV apical sparing is not yet part of the clinical routine, because measurement of LS is time consuming and requires a level of expertise [7], [8]. Kawakami et al. previously revealed that full- and semi-automatic assessment of global longitudinal strain was useful to evaluate LV function in terms of rapidity, reproducibility and clinical implications [24]. However, there were no data about the usefulness of automatic assessment of RapLSI to diagnose ATTR-CM. To our knowledge, this is the first study to compare the feasibility, reproducibility, and clinical implications between full-automatic, semi-automatic and manual assessment of RapLSI.

The greatest strengths of automatic assessment are rapidity and reproducibility [25], [26]. The present study revealed that full-automatic assessment could calculate LS within only 20 s, supports the strength of full-automatic assessment in rapidity. Moreover, semi-automatic assessed LS needed approximately one-third of the time of manual assessment LS. For reproducibility, it is apparent that full-automatic assessment usually yields the same results, when the same images were provided (data not shown). Even in semi-automatic assessment, our present study revealed high intra- and inter-observer reproducibility. These results indicated that high rapidity and reproducibility of full- and semi- automatic assessment could contribute to the wider use of RapLSI to diagnose ATTR-CM in clinical practice.

The present study also revealed some limitations of RapLSI estimated by full-automatic assessment. Manual correction was needed for many patients after the automatic assessment of LS. The diagnostic accuracy of full-automatic assessed RapLSI to predict ATTR-CM was lower than those of semi-automatic assessed RapLSI and of manual assessed RapLSI. These findings suggest that full-automatic assessment of RapLSI is lack feasibility for clinical use to diagnose ATTR-CM. In contrast, there was no significant difference between the diagnostic accuracy of RapLSI estimated by semi-automatic assessment and that estimated by manual assessment. Considering rapidity, reproducibility and diagnostic accuracy, semi-automatic assessment of RapLSI seems to provide a better balance between feasibility and clinical relevance.

Phelan et al. indicated the extremely high diagnostic accuracy of RapLSI to diagnose amyloid cardiomyopathy (sensitivity 93% and specificity 82%) by the cutoff point of RapLSI as 1.00^5^. However, the sensitivity of RapLSI in our present study was relatively lower than it in the previous report. There were many AS patients in our present study. We previously reported that the diagnostic utility of LV apical sparing for ATTR-CM was relatively low in AS patients [27]. These might be one of the reasons of low sensitivity of RapLSI to evaluate ATTR-CM. In addition, several studies revealed the limitation of LV apical sparing to evaluate amyloid cardiomyopathy. We previously reported that half of ATTR-CM did not have LV apical sparing [28]. Kyrouac et al. showed that LV apical sparing had modest discriminating ability for amyloid cardiomyopathy (66% sensitivity and 59% specificity) and several patients in non-amyloid cardiomyopathy group also had LV apical sparing in the real world [29]. These results indicated that LV apical sparing was useful, but not perfect technique to diagnose ATTR-CM. Therefore, we have to diagnose ATTR-CM from various perspectives.

### 4.1 Study limitations

This study had several potential limitations. First, this study was single center retrospective cohort study and it included a small number of patients. Second, some patients had atrial fibrillation in our present study, although it is sometime difficult to evaluate strain analysis in patients with atrial fibrillation. Third, there could be some AL patients in non ATTR-CM group, because we did not perform endocardial biopsy in all enrolled patients.

Despite these limitations, our study is unique and the first to demonstrate the feasibility, reproducibility and clinical applicable means of RapLSI estimated by semi-automatic assessment.

## 5 Conclusion

There was no significant difference between the diagnostic accuracy of RapLSI estimated by semi-automatic assessment and that estimated by manual assessment. Semi-automatic approach using novel automated software was useful to diagnose ATTR-CM in the terms of rapidity, reproducibility and diagnostic accuracy.

## Ethics Approval

This study was approved by the institutional review board and ethics committee of Kumamoto University (Reference number: 1588).

## Source of Funding

This study was supported in part by Grants-in-Aid for Scientific Research (reference number 20 K17087) from the Ministry of Education, Culture, Sports, Science and Technology of Japan, Grant-in-Aid for Scientific Research (reference number 20 K08476) from Japan Society for the Promotion of Science and research grants from Pfizer Japan Inc.

## Registration number

There is no registration number because this study is purely observational study.

## Data Availability statements

The data underlying the research results described in this paper will be shared on reasonable request to the corresponding author.

## Declaration of Competing Interest

The authors declare that they have no known competing financial interests or personal relationships that could have appeared to influence the work reported in this paper.

## Figures and Tables

**Supplementary Fig. 1:**
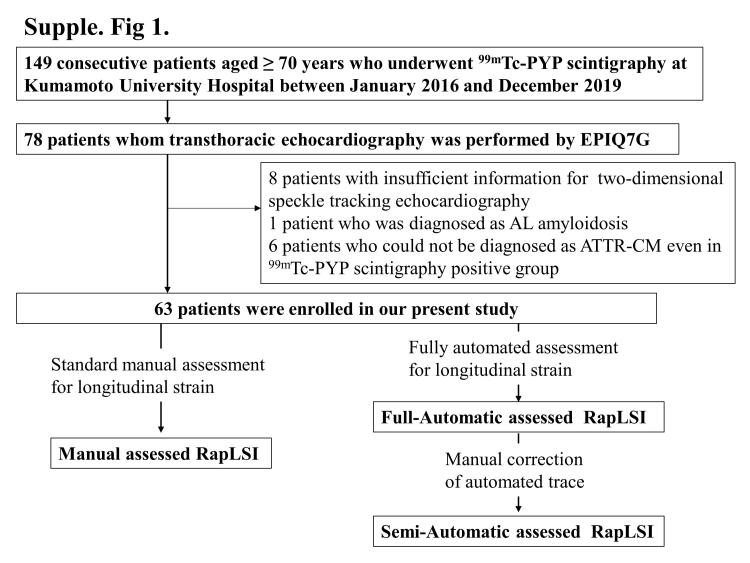


**Supplementary Fig. 2:**