# Supplementary material for: Usefulness of automatic assessment for longitudinal strain to diagnose wild-type transthyretin amyloid cardiomyopathy
Source: Int J Cardiol Heart Vasc. 2023 Jun 22;47:101227. doi: 10.1016/j.ijcha.2023.101227 (PMC10320495; doi:10.1016/j.ijcha.2023.101227)
Supplement: Supplementary Figure legend [file mmc1.docx]

**Supplementary Figure legend**

Supple. Fig. 1. Study flow chart showing selection of the present study population for full-automatic, semi-automatic and manual assessment of RapLSI

RapLSI, relative apical longitudinal strain index

Supple. Fig. 2. The difference of predictive model for ATTR-CM between each assessment. Positive predictive value of ATTR-CM in high RapLSI group were 77%, 100% and 96% and negative predictive value of ATTR-CM in low RapLSI group were 68%, 76% and 77%, when RapLSI was estimated by full-automatic assessment, semi-automatic assessment and manual assessment, respectively.
