# Supplementary material for: Usefulness of automatic assessment for longitudinal strain to diagnose wild-type transthyretin amyloid cardiomyopathy
Source: Int J Cardiol Heart Vasc. 2023 Jun 22;47:101227. doi: 10.1016/j.ijcha.2023.101227 (PMC10320495; doi:10.1016/j.ijcha.2023.101227)
Supplement: Supplemental Table 1 [file mmc2.docx]

**Supplemental table 1. Comparison of clinical characteristics between ATTR-CM group and non ATTR-CM group**

|  | ATTR-CM group  (n=32) | Non ATTR-CM group (n=31) | p value |
| --- | --- | --- | --- |
| Basal clinical characteristics | | | |
| Age, years  Male sex (%)  BMI, kg/m^2^  HT (%)  DM (%)  Dyslipidemia (%)  Smoker (%)  OMI (%)  HD (%)  Chronic Atrial fibrillation (%)  NYHA class ≥ 2 | 77.2±4.9  28 (88)  23.0±2.7  17 (53)  9 (28)  10 (31)  1 (3)  2 (6)  0 (0)  10 (31)  24 (75) | 81.4±6.2  20 (65)  21.4±3.2  17 (55)  7 (23)  13 (42)  3 (10)  0 (0)  2 (6)  8 (26)  19 (61) | <0.01  <0.05  <0.05  0.89  0.61  0.38  0.30  0.16  0.14  0.63  0.24 |
| Laboratory findings | | | |
| Hs-cTnT, ng/ml  BNP, pg/ml  CRP, mg/dl  Hemoglobin level, g/dl  eGFR, ml/min/1.73m^2^ | 0.04 [0.03-0.07]  218 [122-394]  0.07 [0.04-0.20]  13.7±1.9  57.5±11.8 | 0.03 [0.02-0.05]  290 [160-495]  0.12 [0.05-0.77]  11.6±1.8  48.7±21.8 | 0.07  0.21  0.12  <0.01  0.05 |
| TTE findings | | | |
| LAVI, mm  IVSTd, mm  LVPWTd, mm  LVEF, %  E/e’ ratio  Aortic valve stenosis (%)  Mitral valve regurgitation (%)  Mitral valve stenosis (%) | 62.7±21.2  15.1±2.2  15.3±3.0  51.5±11.2  21.6±7.3  5 (16)  2 (6)  0 (0) | 72.3±48.0  12.9±2.7  12.2±1.7  58.6±8.3  20.0±8.8  14 (45)  4 (13)  1 (3) | 0.32  <0.01  <0.01  <0.01  0.43  0.11  0.37  0.31 |

p values were obtained by the student’s t-test, the Mann–Whitney U test and chi-square test.

Abbreviations; BMI, body mass index; HT, hypertension; DM, diabetes mellitus; OMT, old myocardial infarction; HD, hemodialysis; hs-TnT, high-sensitivity cardiac troponin T; BNP, B-type natriuretic peptide; CRP, C reactive protein; eGFR, estimated glomerular filtration rate; TTE, transthoracic echocardiography; LAVI, left atrial volume index; IVSTd, interventricular septum thickness in diastole; LVPWTd, left ventricular posterior wall thickness in diastole; LVEF, left ventricular ejection fraction.
